# Nonlinear optomechanical detection for Majorana fermions *via* a hybrid nanomechanical system

**DOI:** 10.1186/1556-276X-9-166

**Published:** 2014-04-05

**Authors:** Hua-Jun Chen, Ka-Di Zhu

**Affiliations:** 1 Department of Physics and Astronomy, Shanghai Jiao Tong University, Shanghai, China

**Keywords:** Majorana fermions, Nanomechanical resonator, Coherent nonlinear optical spectroscopy

## Abstract

The pursuit for detecting the existence of Majorana fermions is a challenging task in condensed matter physics at present. In this work, we theoretically propose a novel nonlinear optical method for probing Majorana fermions in the hybrid semiconductor/superconductor heterostructure. Our proposal is based on a hybrid system constituted by a quantum dot embedded in a nanomechanical resonator. With this method, the nonlinear optical Kerr effect presents a distinct signature for the existence of Majorana fermions. Further, the vibration of the nanomechanical resonator will enhance the nonlinear optical effect, which makes the Majorana fermions more sensitive to be detected. This proposed method may provide a potential supplement for the detection of Majorana fermions.

## Background

The search for Majorana fermions (MFs) in hybrid nanostructures of condensed matter systems has become an important topic in quantum information processing. Unlike the usual Dirac particles, MFs obey non-Abelian statistics, which will open the potential applications in topological quantum computation [1-3]. In recent years, a number of systems that might host MFs in solid-state scenarios have been proposed. Several typical proposals include atoms trapped in optical lattices [4,5], heterostructures of topological insulators and superconductor [6,7], carbon-based materials [8], p-wave superconductors [9-11], and graphene or graphene-like materials [12]. Beyond these proposals, one promising scheme is to use semiconducting nanowires (such as InAs and InSb nanowires) with strong spin-orbit coupling placed in proximity with a superconductor and biased with an external magnetic field [13,14]. After the prediction that Majorana bound states (MBSs) can be observed in the hybrid semiconductor/superconductor heterostructure, various experiments have indeed reported signatures of MFs in such systems recently [15-20].

Since MFs are their own antiparticles, they are predicted to appear in tunneling spectroscopy experiments as zero-bias peaks [21-23]. Such peaks have been observed in several experiments and have been interpreted as the signatures of MFs [15-19]. Unfortunately, a zero-bias anomaly might also occur under similar conditions due to a Kondo resonance once the magnetic field has suppressed the superconducting gap enough to permit the screening of a localized spin [18,24], and these experiments are not spatially resolved to detect the position of the MFs. Additionally, in many instances, the presence of disorder can also result in spurious zero-bias anomalies even when the system is not topological [25-27]. Except zero-bias conductance peak, the Josephson effect is another signature which can demonstrate Majorana particles in the hybrid semiconductor-superconductor junction [20,28,29]. However, most of the recent experiments proposed and carried out have focused on electrical scheme, and the observation of Majorana signature based on electrical methods still remains a subject of debate. Meanwhile, other effective methods, such as optical technique [30,31], for detecting MFs in the hybrid semiconductor/superconductor heterostructure have received less attention until now.

In recent years, nanostructures such as quantum dots (QDs) and nanomechanical resonators (NRs) have been obtained significant progress in modern nanoscience and nanotechnology. QD, as a simple stationary atom with well optical property [32], lays the foundation for numerous possible applications [33]. On the other hand, NRs are applied to ultrasensitive detection of mechanical signal [34], mass [35,36], mechanical displacements [37], and spin [38] due to their high natural frequencies and large quality factors [39]. Further, the hybrid system where a QD is coupled to the NR also attracts much interest [40-42]. Based on the advantages of QD or NR, several groups propose a scheme for detecting MFs *via* the QD [43-48] or the NR [49] coupled to the nearby MFs. Here, we will propose an optical scheme to detect the existence of MFs in such a hybrid semiconductor/superconductor heterostructure *via* a hybrid QD-NR system.

**Figure 1:**
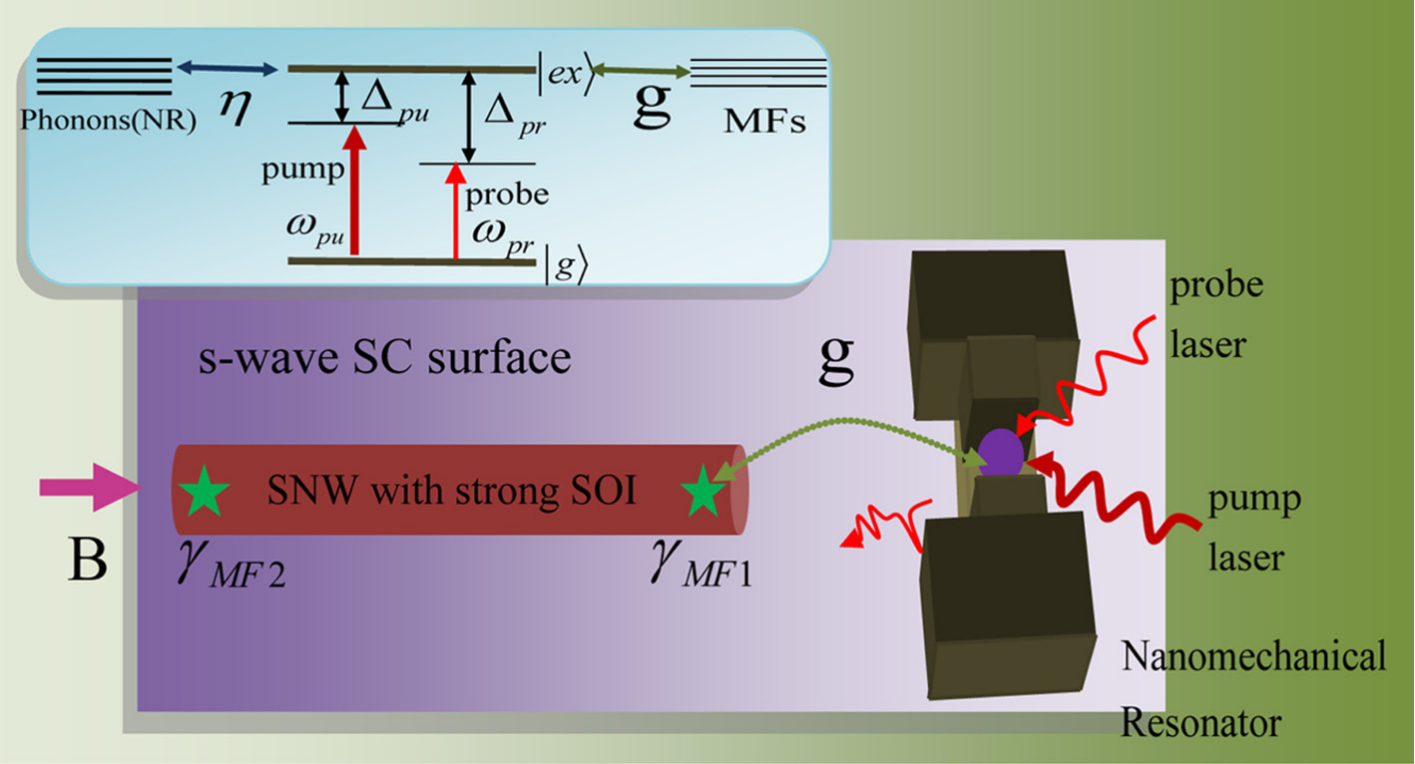
**Sketch of the proposed setup for optically detecting MFs.** An InSb semiconductor nanowire (SNW) with strong spin-orbit interaction (SOI) in an external aligned parallel magnetic field **B** is placed on the surface of a bulk s-wave superconductor (SC). The two green stars at the ends of the nanowire represent a pair of MFs. The nearby MF is coupled to a semiconductor QD embedded in a nanomechanical resonator under a strong pump laser and a weak probe laser simultaneously. The inset is an energy-level diagram of a semiconductor QD coupled to MFs and NR.

In the present article, we consider a scheme closed to that of the recent experiment by Mourik et al. [15]. Compared with zero-bias peaks and the Josephson effect, we adopt an optical pump-probe technique to detect MFs. The nonlinear optical Kerr effect, as a distinct signature for demonstrating the existence of MFs in the hybrid semiconductor/superconductor heterostructure, is the main result of this work. Further, in our system (see Figure 1), the NR as a phononic cavity will enhance the nonlinear optical effect significantly, which makes MFs more sensitive to be detected.

## Model and theory

Figure 1 presents the schematic setup that will be studied in this work. An InSb semiconductor nanowire with spin-orbit coupling in an external aligned parallel magnetic field **B** is placed on the surface of a bulk s-wave superconductor (SC). A MF pair is expected to locate at the ends of nanowire. To detect MFs, we employ a hybrid system in which an InAs semiconductor QD is embedded in a GaAs NR. By applying a strong pump laser and a weak probe laser to the QD simultaneously, one could probe the MFs *via* optical pump-probe technique [30,31].

Benefitting from recent progress in nanotechnology, the quantum nature of a mechanical resonator can be revealed and manipulated in the hybrid system where a single QD is coupled to a NR [40-42]. In such a hybrid system, the QD is modeled as a two-level system consisting of the ground state |*g*〉 and the single exciton state |*ex*〉 at low temperatures [50,51]. The Hamiltonian of the QD can be described as $H_{\text{QD}}=\hbar \omega_{\text{QD}}S^{z}$ with the exciton frequency *ω*_QD_, where *S*^*z*^ is the pseudospin operator. In a structure of the NR where the thickness of the beam is much smaller than its width, the lowest-energy resonance corresponds to the fundamental flexural mode that will constitute the resonator mode [40]. We use a Hamiltonian of quantum harmonic oscillator $H_{m}=\hbar \omega_{m}b^{+}b$ with the frequency *ω*_*m*_ and the annihilation operator *b* of the resonator mode to describe the eigenmode. Since the flexion induces extensions and compressions in the structure [52], this longitudinal strain will modify the energy of the electronic states of QD through deformation potential coupling. Then the coupling between the resonator mode and the QD is described by $\hbar \omega_{m}\eta S^{z}(b^{+}+b)$, where *η* is the coupling strength between the resonator mode and QD [40]. Therefore, the Hamiltonian of the hybrid QD-NR system is $H_{\text{QD-NR}}=\hbar \omega_{\text{QD}}S^{z}+\hbar \omega_{m}b^{+}b+\hbar \omega_{m}\eta S^{z}(b^{+}+b)$.

Since several experiments [15-20] have reported the distinct signatures of MFs in the hybrid semiconductor/superconductor heterostructure *via* electrical methods, we assure that the MFs may exist in these hybrid systems under some appropriate conditions. Based on these experimental results, in the present article, we will try to demonstrate the MFs by using nonlinear optical method. As each MF is its own antiparticle, one can introduce a MF operator *γ*_MF_ such that $\gamma_{\text{MF}}^{‡}=\gamma_{\text{MF}}$ and $\gamma_{\text{MF}}^{2}=1$ to describe MFs. Supposing the QD couples to *γ*_MF1_, the Hamiltonian of the hybrid system [43-46] is $H_{\text{MF}}=\mathrm{i\hbar}\omega_{\text{MF}}\gamma_{\text{MF1}}\gamma_{\text{MF2}}/2+\mathrm{i\hbar g}(S^{-}-S^{+})\gamma_{\text{MF1}}$, where *S*^±^ are the pseudospin operators. To detect the existence of MFs, it is helpful to switch from the Majorana representation to the regular fermion one *via* the exact transformation $\gamma_{\text{MF1}}=f_{M}^{+}+f_{M}$ and $\gamma_{\text{MF2}}=i(f_{M}^{+}-f_{M})$. *f*_*M*_ and $f_{M}^{+}$ are the fermion annihilation and creation operators obeying the anti-commutative relation $\left\{f_{M},f_{M}^{+}\right\}=1$. Accordingly, in the rotating wave approximation [53], *H*_*M*_ can be rewritten as $H_{\text{MF}}=\hbar \omega_{\text{MF}}(f_{M}^{+}f_{M}-1/2)+\mathrm{i\hbar g}(S^{-}f_{M}^{+}-S^{+}f_{M})$, where the first term gives the energy of MF at frequency *ω*_MF_, and $\hbar \omega_{\text{MF}}=\varepsilon_{\text{MF}}∼e^{-l/\xi }$ with the wire length (*l*) and the superconducting coherent length (*ξ*). This term is small and can approach zero as the wire length is large enough. The second term describes the coupling between the right MF and the QD with coupling strength *g*, where the coupling strength depends on the distance between the hybrid QD-NR system and the hybrid semiconductor/superconductor heterostructure. Compared with electrical detection scheme which the QD is coupled to MF *via* the tunneling, here in our optical scheme, the exciton-MF coupling is mainly due to the dipole-dipole interaction. Since in current experiments the distance between QD and MF can be adjusted to locate the distance by about several tens of nanometers. In this case, the tunneling between the QD and MF can be neglected. It should be also noted that the term of non-conservation for energy, i.e. $\mathrm{i\hbar g}(S^{-}f_{M}-S^{+}f_{M}^{+})$, is generally neglected. We have made the numerical calculations (not shown in the following figures) and shown that the effect of this term is too small to be considered in our theoretical treatment, especially for calculating the nonlinear optical properties of the QD.

The optical pump-probe technology includes a strong pump laser and a weak probe laser [54], which provides an effective way to investigate the light-matter interaction. Based on the optical pump-probe scheme, the linear and nolinear optical effects can be observed *via* the probe absorption spectrum. Xu et al. [30] have obtained coherent optical spectroscopy of a strongly driven quantum dot without a nanomechanical resonator. Recently, this optical pump-probe scheme has also been demonstrated experimentally in a cavity optomechanical system [31]. In terms of this scheme, we apply a strong pump laser and a weak probe laser to the QD embedded in the NR simultaneously. The Hamiltonian of the QD coupled to the pump laser and probe laser is given by [54]$H_{\text{QD-L}}=-\text{µ}E_{\text{pu}}(S^{+}e^{-i\omega_{\text{pu}}t}+S^{-}e^{i\omega_{\text{pu}}t})-\text{µ}E_{\text{pr}}(S^{+}e^{-i\omega_{\text{pr}}t}+S^{-}e^{i\omega_{\text{pr}}t})$, where µ is the dipole moment of the exciton, *ω*_pu_ (*ω*_pr_) is the frequency of the pump (probe) laser, and *E*_pu_ (*E*_pr_) is the slowly varying envelope of the pump (probe) laser. Therefore, one can obtain the total Hamiltonian of the hybrid system as *H*=*H*_QD-NR_+*H*_MBS_+*H*_QD-L_.

According to the Heisenberg equation of motion and introducing the corresponding damping and noise terms, in a rotating frame at the pump laser frequency *ω*_pu_, we derive the quantum Langevin equations of the coupled system as follows:

(1) $$\begin{array}{c} \;{\dot{S}}^{z}=-\;\Gamma_{1}\;\;\left(S^{z}\;+\;\frac{1}{2}\right)\;+\;i\Omega_{\text{pu}}\;\left(S^{+}\;-\;S^{-}\right)\;-\;g\;\left(S^{-}f_{M}^{+}\;+\;S^{+}f_{M}\right)\\ \;+\frac{i\text{µ}E_{\text{pr}}}{\hbar }(S^{+}e^{-\mathrm{i\delta t}}-S^{-}e^{\mathrm{i\delta t}}), \end{array}$$

(2) $$\begin{array}{c} \;{\dot{S}}^{-}=-[i(\Delta_{\text{pu}}+\omega_{m}\mathrm{\eta N})+\Gamma_{2}]S^{-}-\frac{2i\text{µ}E_{\text{pr}}}{\hbar }e^{-\mathrm{i\delta t}}S^{z}\\ \;+2({\text{gf}}_{M}-i\Omega_{\text{pu}})S^{z}+{\hat{F}}_{\text{in}}(t), \end{array}$$

(3) $$\begin{array}{c} \;{\dot{f}}_{M}=-(i\Delta_{\text{MF}}+\kappa_{\text{MF}}/2)f_{M}+gS^{-}+{\hat{\xi }}_{\text{MF}}(t), \end{array}$$

(4) $$\begin{array}{c} \;\ddot{N}+\gamma_{m}\dot{N}+\omega_{m}^{2}N=-2\omega_{m}^{2}\eta S^{z}+\hat{\xi }(t), \end{array}$$

where *N*=*b*^+^+*b*. *Γ*_1_ (*Γ*_2_) is the exciton relaxation rate (dephasing rate), *κ*_MF_ (*γ*_*m*_) is the decay rate of the MF (nanomechanical resonator). *Δ*_pu_=*ω*_QD_-*ω*_pu_ is the detuning of the exciton frequency and the pump frequency, $\Omega_{\text{pu}}=\text{µ}E_{\text{pu}}/\hbar$ is the Rabi frequency of the pump field, and *δ*=*ω*_pr_-*ω*_pu_ is the probe-pump detuning. *Δ*_MF_=*ω*_MF_-*ω*_pu_ is the detuning of the MF frequency and the pump frequency. ${\hat{F}}_{\text{in}}(t)$ is the *δ*-correlated Langevin noise operator, which has zero mean $\langle {\hat{F}}_{\text{in}}(t)\rangle =0$ and obeys the correlation function $\langle {\hat{F}}_{\text{in}}(t){\hat{F}}_{\text{in}}^{‡}(t^{{}^{\prime }})\rangle =\delta (t-t^{{}^{\prime }})$. The resonator mode is affected by a Brownian stochastic force with zero mean value, and $\hat{\xi }(t)$ has the correlation function $\langle {\hat{\xi }}^{+}(t)\hat{\xi }(t^{{}^{\prime }})\rangle =\frac{\gamma_{m}}{\omega_{m}}\int \frac{\mathrm{d\omega}}{2\pi }\omega e^{-\mathrm{i\omega}(t-t^{{}^{\prime }})}[1+\coth(\frac{\hbar \omega }{2\kappa_{B}T})]$, where *k*_*B*_ and *T* are the Boltzmann constant and the temperature of the reservoir, respectively. MFs have the same correlation relation as the resonator mode. Actually, we have neglected the regular fermions (i.e. normal electrons) in the nanowire that interact with the QD in the above discussion. To describe the interaction between the normal electrons and the QD, we use the tight-binding Hamiltonian of the whole wire as [55,56]$H_{\text{QD-e}}=\hbar \omega_{\text{QD}}S^{z}+\hbar \underset{k}{\sum }\omega_{k}c_{k}^{+}c_{k}+\hbar \zeta \underset{k}{\sum }(c_{k}^{+}S^{-}+S^{+}c_{k})$, where *c*_*k*_ and $c_{k}^{‡}$ are the regular fermion annihilation and creation operators with energy *ω*_*k*_ and momentum ℏk obeying the anti-commutative relation $\left\{c_{k},c_{k}^{‡}\right\}=1$ and *ζ* is the coupling strength between the normal electrons and QD (here, for simplicity, we have neglected the *k*-dependence of *ζ* as in [57]).

To go beyond weak coupling, the Heisenberg operator can be rewritten as the sum of its steady-state mean value and a small fluctuation with zero mean value: $S^{z}=S_{0}^{z}+\delta S^{z}$, $S^{-}=S_{0}^{-}+\delta S^{-}$, *f*_*M*_=*f*_*M*0_+*δf*_*M*_ and *N*=*N*_0_ +*δN*. Since the driving fields are weak, but classical coherent fields, we will identify all operators with their expectation values, and drop the quantum and thermal noise terms [31]. Simultaneously, inserting these operators into the Langevin equations (Equations 1 to 4) and neglecting the nonlinear term, we can obtain two equation sets about the steady-state mean value and the small fluctuation. The steady-state equation set consisting of *f*_*M*0_, *N*_0_ and $S_{0}^{-}$ is related to the population inversion ($w_{0}=2S_{0}^{z}$) of the exciton which is determined by $\Gamma_{1}(w_{0}+1)[(\Delta_{\text{MF}}^{2}+\kappa_{\text{MF}}^{2}/4)(\Delta_{\text{pu}}^{2}+\Gamma_{2}^{2}+\omega_{m}^{2}\eta^{4}w_{0}^{2}-2\omega_{m}\Delta_{\text{pu}}\eta^{2}w_{0})+g^{2}w_{0}^{2}(g^{2}-2\omega_{m}\Delta_{\text{MF}}\eta^{2}+2\Delta_{\text{pu}}\Delta_{\text{MF}}-\Gamma_{2}\kappa_{\text{MF}})]+4\Omega_{\text{pu}}^{2}w_{0}\Gamma_{2}(\Delta_{\text{MF}}^{2}+\kappa_{\text{MF}}^{2}/4)=0$. For the equation set of small fluctuation, we make the ansatz [54]$\langle \delta S^{z}\rangle =S_{+}^{z}e^{-\mathrm{i\delta t}}+S_{-}^{z}e^{\mathrm{i\delta t}}$, 〈*δS*^-^〉=*S*_+_*e*^-*iδt*^+*S*_-_*e*^*iδt*^, 〈*δf*_*M*_〉=*f*_*M*+_*e*^-*iδt*^+*f*_*M*-_*e*^*iδt*^, and 〈*δN*〉=*N*_+_*e*^-*iδt*^+*N*_-_*e*^*iδt*^. Solving the equation set and working to the lowest order in *E*_pr_ but to all orders in *E*_pu_, we can obtain the nonlinear optical susceptibility as $\chi_{\text{eff}}^{\left(3\right)}(\omega_{\text{pr}})=\text{µ}S_{-}(\omega_{\text{pr}})/(3E_{\text{pu}}^{2}E_{\text{pr}})=\Sigma_{3}\chi^{\left(3\right)}(\omega_{\text{pr}})$, where $\Sigma_{3}={\text{µ}}^{4}/(3\hbar^{3}\Gamma_{2}^{3})$ and *χ*^(3)^(*ω*_pr_) is given by

(5) $$\chi^{\left(3\right)}(\omega_{\text{pr}})=\frac{(d_{2}^{*}+h_{4}d_{1}^{*})d_{3}h_{6}-{\text{iw}}_{0}d_{3}h_{4}}{(h_{4}h_{5}d_{3}d_{1}^{*}-d_{4}d_{2}^{*})\Omega_{\text{pu}}^{2}}\Gamma_{2}^{3},$$

where *b*_1_=*g*/[*i*(*Δ*_MF_-*δ*)+*κ*_MF_/2], *b*_2_=*g*/[ *i*(*Δ*_MF_+*δ*)+*κ*_MF_/2], $b_{3}=2\omega_{m}^{2}\eta /(\delta^{2}+\mathrm{i\delta}\gamma_{m}-\omega_{m}^{2})$, $h_{4}=[(i\Omega_{\text{pu}}-{\text{gf}}_{M0})-{\text{gS}}_{0}^{-}b_{1}^{*}]/(\Gamma_{1}+\mathrm{i\delta})$, $h_{5}=-[\;(i\Omega_{\text{pu}}+{\text{gf}}_{M0}^{*})+{\text{gS}}_{0}^{-*}b_{2}]/(\Gamma_{1}+\mathrm{i\delta})$, $h_{6}={\text{iS}}_{0}^{-}/(\Gamma_{1}+\mathrm{i\delta})$, $d_{1}=2({\text{gf}}_{M0}-i\Omega_{\text{pu}})-i\omega_{m}\eta S_{0}^{-}b_{3}$, *d*_2_=*i*(*Δ*_pu_-*δ*+*ω*_*m*_*ηN*_0_)+*Γ*_2_-*gb*_1_*w*_0_-*d*_1_*h*_2_, $d_{3}=2({\text{gf}}_{M0}-i\Omega_{\text{pu}})-i\omega_{m}\eta S_{0}^{-}b_{3}^{*}$, *d*_4_=*i*(*Δ*_pu_+*δ*+*ω*_*m*_*ηN*_0_)+*Γ*_2_-*gb*_2_*w*_0_-*d*_3_*h*_5_ (where *O*^∗^ indicates the conjugate of *O*). The quantum Langevin equations of the normal electrons coupled to the QD have the same form as MFs; therefore, we omit its derivation and only give the numerical results in the following.

## Numerical results and discussions

For illustration of the numerical results, we choose the realistic hybrid systems of the coupled QD-NR system [40] and the hybrid semiconductor/superconductor heterostructure [15-17,20]. For an InAs QD in the coupled QD-NR system, the exciton relaxation rate *Γ*_1_=0.3 GHz, the exciton dephasing rate *Γ*_2_=0.15 GHz. The physical parameters of GaAs nanomechanical resonator are (*ω*_*m*_, *m*, *Q*)=(1.2 GHz, 5.3×10^-15^ g, 3×10^4^), where *m* and *Q* are the effective mass and quality factor of the NR, respectively. The decay rate of the NR is *γ*_*m*_= *ω*_*m*_/*Q*=4×10^-5^ GHz. The coupling strength between quantum dot and nanomechanical resonator is *η*=0.06. For MFs in the the hybrid semiconductor/superconductor heterostructure, there are no experimental values for the lifetime of the MFs and the coupling strength between the exciton and MFs in the recent literature. However, according to a few experimental reports [15-17], it is reasonable to assume that the lifetime of the MFs is *κ*_MF_=0.1 MHz. Since the coupling strength between the QD and nearby MFs is dependent on their distance, we also expect the coupling strength *g*=0.03 GHz *via* adjusting the distance between the QD-NR hybrid structure and the nanowire.

**Figure 2:**
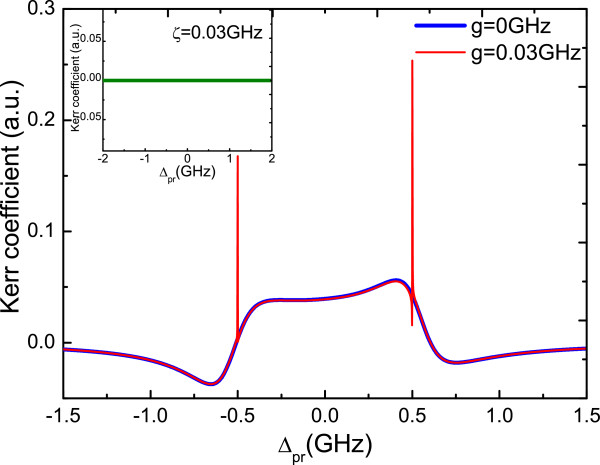
**Optical Kerr coefficient as function of probe detuning*Δ***_**pr**_** with two different QD-MF coupling strengths.** The inset shows the result for the normal electrons in the nanowire that couple to the QD at the coupling strength *ζ*=0.03 GHz. The parameters used are *Γ*_1_=0.3 GHz, *Γ*_2_=0.15 GHz, *η*=0, *γ*_*m*_=4×10^-5^ GHz, *ω*_*m*_=1.2 GHz, *κ*_MF_=0.1 MHz, $\Omega_{\text{pu}}^{2}=0.01$ GHz^2^, *Δ*_MF_=-0.5 GHz, and *Δ*_pu_=0.5 GHz.

Firstly, we consider the case that there is no coupling between the QD and NR (*η*=0), i.e. only a single QD is coupled to the nanowire. Figure 2 plots the optical Kerr coefficient *Re*(*χ*^(3)^) as a function of the probe detuning *Δ*_pr_. In Figure 2, the blue curve indicates the nonlinear optical spectrum without the QD-MF coupling, and the red one shows the result with the QD-MF coupling *g*=0.03 GHz. It is obvious that when the MFs are presented at the ends of the nanowire, the two sharp sideband peaks will appear in the optical Kerr spectrum of the QD. The physical origin of this result is due to the QD-MF coherent interaction, which makes the resonant enhancement of the optical Kerr effect in the QD. This result also implies that the sharp peaks in the nonlinear optical spectrum may be the signature of MFs at the ends of the nanowire. Because there also includes normal electrons in the nanowire, in order to determine whether or not this signature (i.e. the sharp peaks) is the true MFs, we plot the inset of Figure 2, which uses the tight binding Hamiltonian to describe the normal electrons. In the figure, the parameters of normal electrons are chosen the same as MFs so that we can compare with the case of MFs. From the figure, we can observe that there is no sharp peak and only a nearly zero line in the spectrum (see the green line in the inset). This result demonstrates that the coupling between the QD and the normal electrons in the nanowire can be neglected in our theoretical treatment. In this case, one may utilize the optical Kerr effect in QD to detect the existence of MFs provided that the QD is close enough to the ends of the nanowire.

**Figure 3:**
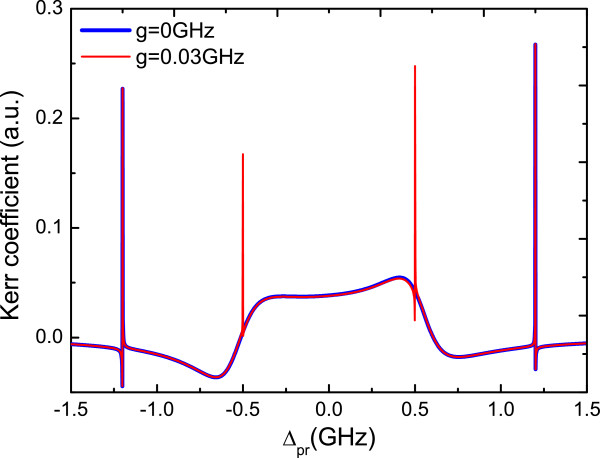
**The optical Kerr coefficient as a function of the probe detuning*Δ***_**pr**_** for*η*=0*.*06.** The other parameters used are the same as Figure 2.

Secondly, we turn on the coupling to the NR (*η*≠0) and then plot the optical Kerr coefficient as a function of probe detuning *Δ*_pr_ for *η*=0.06 as shown in Figure 3. Taking the coupling between the QD and NR into consideration, the other two sharp peaks located at ±*ω*_*m*_ will also appear. The red and blue curves correspond to the optical Kerr coefficient with and without the QD-MF coupling, respectively. Without the QD-MF coupling, the two sharp peaks locate at the resonator frequency of nanomechanical resonator induced by its vibration, i.e. two peaks are at *Δ*_pr_=±1.2 GHz as shown in Figure 3. The physical origin of this result is due to mechanically induced coherent population oscillation (MICPO), which makes quantum interference between the resonator and the beat of the two optical fields *via* the QD when the probe-pump detuning is equal to the resonator frequency [58]. Turning on the QD-MF coupling, in addition to two sharp peaks located at ±1.2 GHz, the other two sideband peaks induced by the QD-MF coupling appear at *Δ*_pr_=±0.5 GHz simultaneously.

**Figure 4:**
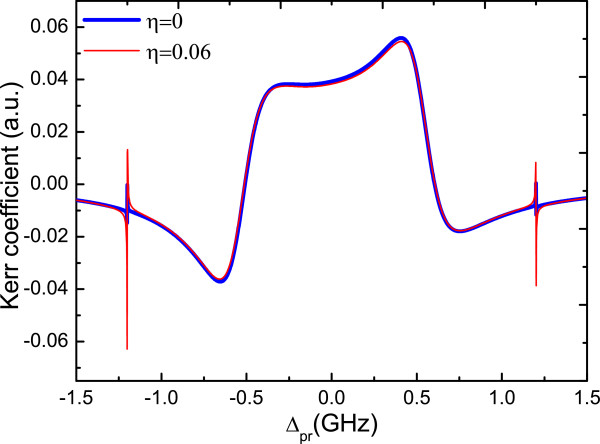
**Optical Kerr coefficient as a function of probe detuning*Δ***_**pr**_** with*η*=0 and*η*=0*.*06.***g*=0.03 GHz and *Δ*_MF_=-1.2 GHz. The other parameters used are the same as Figure 2.

To illustrate the advantage of the NR in our system, we adjust the detuning *Δ*_MF_=-0.5 GHz to *Δ*_MF_=-1.2 GHz, in this case, the location of the two sideband peaks induced by the QD-MF coupling coincides with the two sharp peaks induced by the vibration of NR, so the NR is resonant with the coupled QD-MF system and makes the coherent interaction of QD-MF more strong. Figure 4 gives the result of the optical Kerr coefficient as a function of probe detuning with or without the QD-NR coupling for the QD-MF coupling *g*=0.03 GHz. The blue and red curves correspond to *η*=0 and *η*=0.06, respectively. It is obvious that the role of NR is to narrow and to increase the optical Kerr effect. In this case, the NR as a phonon cavity will enhance the sensitivity for detecting MFs.

## Conclusion

We have proposed a nonlinear optical method to detect the existence of Majorana fermions in semiconductor nanowire/superconductor hybrid structure *via* a single quantum dot coupled to a nanomechanical resonator. The optical Kerr effect may provide another supplement for detecting Majorana fermions. Due to the nanomechanical resonator, the nonlinear optical effect becomes much more significant and then enhances the detectable sensitivity of Majorana fermions. Finally, we hope that our proposed scheme can be realized experimentally in the future.

## Competing interests

The authors declare that they have no competing interests.

## Authors’ contributions

HJC finished the main work of this paper, including deducing the formulas, plotting the figures, and drafting the manuscript. KDZ conceived of the idea, participated in the discussion, and provided some useful suggestion. Both authors are involved in revising the manuscript. Both authors read and approved the final manuscript.
